# Collective orientational order and phase behavior of a discotic liquid crystal under nanoscale confinement†

**DOI:** 10.1039/c8na00308d

**Published:** 2018-12-03

**Authors:** Arda Yildirim, Kathrin Sentker, Glen Jacob Smales, Brian Richard Pauw, Patrick Huber, Andreas Schönhals

**Affiliations:** Bundesanstalt für Materialforschung und-prüfung (BAM) Unter den Eichen 87 12205 Berlin Germany Andreas.Schoenhals@bam.de +49 30/8104-3384; Institut für Materialphysik und-technologie, Technische Universität Hamburg Eißendorfer Str. 42 21073 Hamburg Germany

## Abstract

The phase behavior and molecular ordering of hexakishexyloxy triphenylene (HAT6) DLCs under cylindrical nanoconfinement are studied utilizing differential scanning calorimetry (DSC) and dielectric spectroscopy (DS), where cylindrical nanoconfinement is established through embedding HAT6 into the nanopores of anodic aluminum oxide (AAO) membranes, and a silica membrane with pore diameters ranging from 161 nm down to 12 nm. Both unmodified and modified pore walls were considered. In the latter case the pore walls of AAO membranes were chemically treated with *n*-octadecylphosphonic acid (ODPA) resulting in the formation of a 2.2 nm thick layer of grafted alkyl chains. Phase transition enthalpies decrease with decreasing pore size, indicating that a large proportion of the HAT6 molecules within the pores has a disordered structure, which increases with decreasing pore size for both pore walls. In the case of the ODPA-modification, the amount of ordered HAT6 is increased compared to the unmodified case. The pore size dependencies of the phase transition temperatures were approximated using the Gibbs–Thomson equation, where the estimated surface tension is dependent on the molecular ordering of HAT6 molecules within the pores and upon their surface. DS was employed to investigate the molecular ordering of HAT6 within the nanopores. These investigations revealed that with a pore size of around 38 nm, for the samples with the unmodified pore walls, the molecular ordering changes from planar axial to homeotropic radial. However, the planar axial configuration, which is suitable for electronic applications, can be successfully preserved through ODPA-modification for most of the pore sizes.

## Abstract 2

The phase behavior and molecular ordering of hexakishexyloxy triphenylene (HAT6) DLCs under cylindrical nanoconfinement, for nanopore diameters ranging from 161 nm down to 12 nm, are studied utilizing differential scanning calorimetry (DSC) and dielectric spectroscopy (DS).

## Graphical abstract

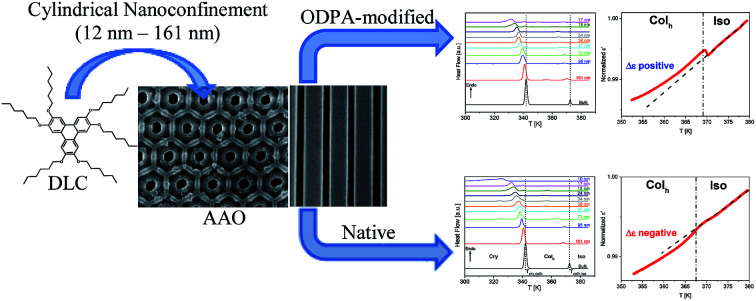

## Introduction

Since the discovery of discotic liquid crystals (DLCs), consisting of molecules having disk-like rigid aromatic cores connected *via* flexible alkyl chains, their intrinsic properties have been extensively investigated to uncover their fundamental properties and potential for applications.^1,2^ Research on DLCs over the last few decades has shown that they can be considered promising materials for use in electronic applications as they exhibit one-dimensional high charge mobility along the axis of the column in the columnar mesophase.^3–6^ The hexagonal columnar mesophase is commonly formed by DLCs, between plastic crystalline and isotropic phases as the molecules can self-organize and stack into columns, which is driven by the favorable π–π interaction between the aromatic cores. Hence, the self-assembly results in favorable charge transport along the axis of the column due to the delocalization of π-electrons. As the alkyl chains fill the intercolumnar space and act as an insulator, the columns can be considered as isolated, one-dimensional, conducting nanowires.^7–9^ This makes DLCs promising materials for electronic applications such as in organic field effect transistors (OFETs), organic light emitting diodes (OLEDs) and organic photovoltaics (OPVs).^3,6,10^

DLCs can also be nanoconfined into a specific geometry, *i.e.* as thin films or in cylindrical nanochannels, in order to optimize their use in electronic applications. Metal oxide membranes with a parallel alignment of cylindrical nanopores and narrow pore size distribution can be produced by electrochemical etching. Confining DLCs into the cylindrical nanopores of metal oxide membranes has gained much interest. This is due to the fact that investigations on such confined systems can address fundamental properties such as phase behavior and glass transition dynamics, whilst also helping to shed light upon the structure–property relationship of soft matter under confinement.^8,9^ Moreover, from an application stand point, it opens up the possibility to prepare nanowires through dissolution of the host membrane.^11^

Nanoconfinement influences the properties of DLCs like for other soft mater systems^12^ which have to be revealed for applications at the nanoscale. The phase behavior under confinement and the molecular ordering of DLCs within pore structures are two of the main properties defining their applications in nanotechnology.^3^ Hence, several investigations have been carried out to elucidate the phase behavior and molecular ordering of DLCs confined within the nanopores of metal oxide membranes.^7–9,13–15^ Compared to bulk DLCs, a decrease in phase transition temperatures is observed alongside the formation of additional structures under confinement that have been reported for pyrene- and triphenylene-based DLCs.^8,9,14^ On the other hand, controlling the molecular ordering within pore structures is highly desirable for improving application relevant properties such as conductivity and light harvesting abilities. Hence, aligning the columns of DCLs parallel to the axis of the pores is therefore crucial to their success in specific applications.

**Fig. 1:**
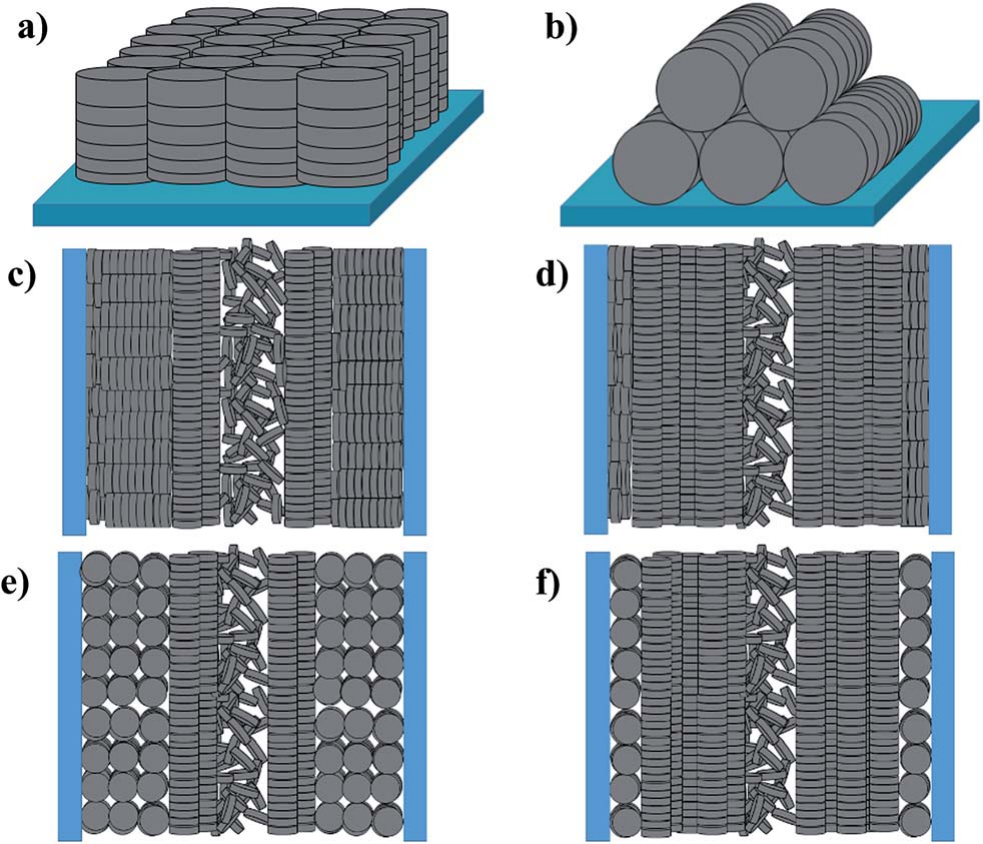
Types of molecular ordering of discotic liquid crystals. (a) Homeotropic (face-on) alignment and (b) planar (edge-on) alignment on a free surface. (c) Homeotropic radial configuration, (d) planar axial configuration, (e) planar radial configuration, and (f) another planar axial configuration dominating configuration type inside a cylindrical channel with a disordered isotropic core.

Planar (edge-on anchoring) and homeotropic (face-on anchoring) alignments are the two possible types of molecular ordering of DLC molecules with respect to a flat surface (Fig. 1a and b). These two alignments describe the ordering in thin films of DLCs and DLCs confined between two parallel solid substrates.^16^ However, when placed under confinement within cylindrical pores, the molecular ordering can become complicated, with confined molecules possessing multiple types of ordering, and thus, describing this ordering within cylindrical pores solely as planar or homeotropic may be insufficient. Hence, additional directional characterization terms such as ‘axial’ and ‘radial’ should be used alongside the anchoring type for the clarity. It has been shown that planar axial, planar radial (circular concentric)^17,18^ and homeotropic radial (logpile)^19^ configurations are the predominant forms of ordering within pore structures (Fig. 1c–f). There is a competition between these configurations, caused by different interfacial tensions observed between air/liquid crystal, liquid crystal/pore walls and liquid crystal/liquid crystal. The dominating type defines the configuration for the overall system.

Among the above-mentioned types of configurations found within the pores, only the axial configuration is suitable for electronic applications. Studies have shown that axial configurations are scarcely observed, whereas homeotropic radial and planar radial configurations are more commonly observed for DLCs confined within the nanopores of metal oxide membranes.^7,14,15,17–19^ However, a dominating axial configuration might be obtained through chemical modification of the pore surface.^7^ Besides the surface modifications changing the host–guest interactions, an appropriate choice of the host pore size with respect to that of the guest DLC can lead to better configurational control. Recently, Zhang *et al.*^18^ reported that the dominating ordering type changes with pore size.^18,19^ Furthermore, they observed a transition from a planar radial configuration to an axial configuration with increasing columnar rigidity of the DLC.^18^ The column rigidity increases with aromatic core size, and hence, it can be tuned to obtain a desired configuration through an appropriate choice of the DLC.

In this study, the effect of cylindrical confinement on the phase behavior and molecular ordering of 2,3,6,7,10,11-hexakis[hexyloxy]triphenylene (HAT6), a triphenylene-based DLC, confined within nanochannels is revealed. Anodic aluminum oxide (AAO) and silica membranes with varying pore diameters, 161 nm down to 12 nm, were used as confining hosts. Some preliminary data for only four pore sizes have been reported for the same system.^8^ However, here a broader pore size range is covered, and therefore a better understanding of the confinement effect on the phase behavior is obtained. This is also due to the fact that the membranes are characterized in detail. Furthermore, it is aimed to obtain an axial ordering or to increase the degree of axial ordering by chemical modification of the pore walls. The phase behavior was explored by differential scanning calorimetry (DSC) allowing the detection of small changes in the phase behavior. Dielectric spectroscopy was demonstrated as a powerful method to monitor molecular ordering within the pores.^15^ Here, we also investigate the collective orientational order, corresponding to dominating molecular configuration, by dielectric spectroscopy.

## Experimental section

### Materials

**Fig. 2:**
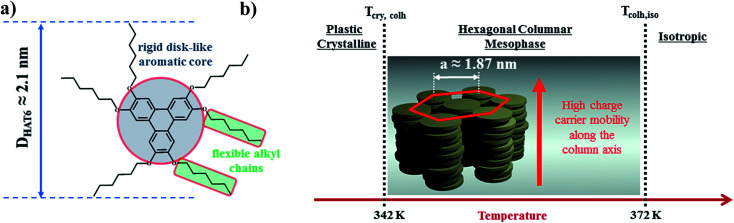
(a) Chemical structure of 2,3,6,7,10,11-hexakis[hexyloxy]triphenylene (HAT6). *D*_HAT6_ is the diameter of HAT6 molecules, reported to be *ca.* 2.1 nm.^20^ (b) Phases formed by HAT6 and HAT6 molecules in the hexagonal columnar liquid crystalline phase. *a* is the hexagonal lattice parameter, reported to be *ca.* 1.87 nm.^19,31^

2,3,6,7,10,11-Hexakis[hexyloxy]triphenylene (HAT6) was purchased from Synthon Chemicals (Bitterfeld, Germany, CAS-no.: 70351-86-9) and used as received. The molecular weight was estimated to be 829.24 g mol^−1^ by MALDI-TOF MS.^31^ According to the producer its purity is at least 98%. At room temperature HAT6 appears as white crystals. The chemical structure of HAT6 (sum formula C_54_H_84_O_6_) and a schematic illustration of its molecular organization and the thermotropic phases for the bulk are shown in Fig. 2. At low temperatures, HAT6 forms a plastic crystalline phases (Cry). Upon further heating the Cry phase, it forms a hexagonal columnar mesophase (Col_h_). In the Col_h_ phase, the HAT6 molecules stack up in columns which are arranged in a hexagonal lattice. With further heating the Col_h_ phase, it undergoes a clearing transition to a more or less isotropic liquid state (Iso).

Disk shaped anodic aluminum oxide (AAO) membranes with a variety of pore diameters, thicknesses and porosities were purchased from Smart Membranes GmbH (Halle, Germany) and InRedox (Longmont, USA). Silica membranes having a pore diameter of *ca.* 12 nm were prepared by electrochemical anodic etching. A highly p-doped 〈100〉 silicon wafer with a resistivity of *R* = 0.01–0.02 Ω cm was used. As an electrolyte solution a mixture of 40 vol% hydrofluoric acid and 60 vol% ethanol was used with an etching current density of 13.3 mA cm^−2^ applied for 8 h.^21^

The porosities and pore diameters of the membranes were characterized by using volumetric N_2_-sorption isotherms at a standard temperature of 77 K. Volumetric N_2_-sorption experiments were carried out by using an Autosorb iQ Quantachrome Instruments gas sorption system. The determined pore sizes and porosities (number of pores per unit area) are given in Table 1 according to the specifications of the producers.

**Table 1:** Properties of the nanoporous membranes

Membrane	Producer's values			Determined values		Producer
	Pore depth (μm)	Pore size, *d* (nm)	Porosity (%)	Pore size, *d* (nm)	Porosity (%)	
Silicon	—	—	—	11.8 ± 0.2	61.2 ± 0.2	—
AAO	80	25	10	16.7 ± 0.6	16.0 ± 1.0	Smart Membranes
AAO	100	20	12	17.8 ± 1.8	19.4 ± 1.6	InRedox
AAO	80	30	30	24.3 ± 0.2	28.7 ± 1.0	Smart Membranes
AAO	80	40^a^	34	34.2 ± 0.7	44.0 ± 0.2	Smart Membranes
AAO	80	40^b^	10	37.8 ± 0.7	15.8 ± 0.9	Smart Membranes
AAO	80	50	15	47.3 ± 0.3	21.5 ± 0.2	Smart Membranes
AAO	80	80	35	72.9 ± 3.1	36.4 ± 1.6	Smart Membranes
AAO	100	120	12	95.0 ± 13.0	7.6 ± 0.6	InRedox
AAO	80	180	10	161.1 ± 9.7	13.8 ± 1.7	Smart Membranes

a Etched in oxalate acid.

b Etched in sulfuric acid.

**Fig. 3:**
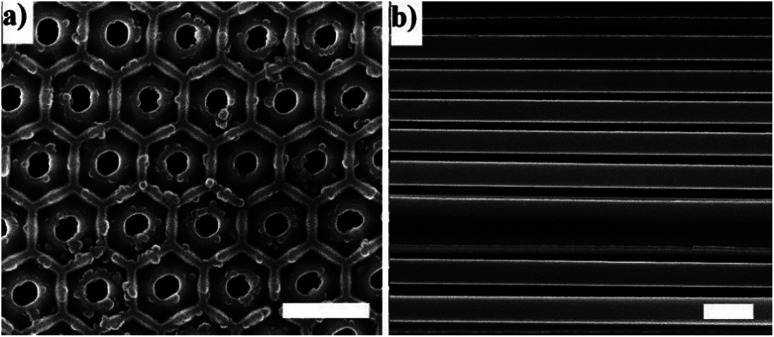
Scanning electron microscopy images (Zeiss Gemini Supra 40) of an AAO membrane with a pore size of 161 nm. (a) The top view of the membrane. (b) The side view of the edge broken membrane. The white scale bars are 600 nm.

The membranes have cylindrical hexagonal ordered pores which are open at both sides. The cylindrical pores are parallel to each other and perpendicular to the surface of the disk-shaped membranes. Fig. 3 demonstrates that the pore distribution of the membranes is narrow, and the pores have an almost parallel arrangement.

### AAO surface modification

Experiments were carried out on both uncoated and coated pore walls. In the latter case the pore walls of the AAO membranes were chemically modified with *n*-octadecylphosphonic acid (C_18_H_39_O_3_P; ODPA) following the procedure reported in the literature.^22,23^ ODPA was purchased from Alfa Aesar and used as received.

**Fig. 4:**
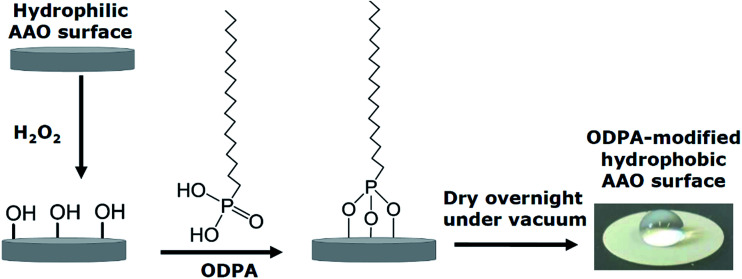
Schematic illustration of the surface modification of the AAO membranes with ODPA. The photo on the right shows a water droplet on a modified membrane to visualize the hydrophobicity of the modified membrane.

A scheme of the modification process is given in Fig. 4. First, the pore walls of the AAO membranes were activated with 30% aqueous H_2_O_2_ solution for 2 h at 45 °C, and then dried at 120 °C for 15 minutes. The pore wall activated membranes were immersed into a 4 mM solution of ODPA in a *n*-heptane/isopropyl alcohol solution (volume ratio of 5 : 1) for 48 h at 25 °C. The membranes were then washed several times and sonicated for 15 minutes with the *n*-heptane/isopropyl alcohol solution to remove any physically absorbed ODPA. The sonicated samples were then washed several times with the *n*-heptane/isopropyl alcohol solution, and then with acetone before being left to dry overnight under vacuum at room temperature. The modification of the pore walls was confirmed by FTIR, as given in ESI, Fig. S1.†

**Fig. 5:**
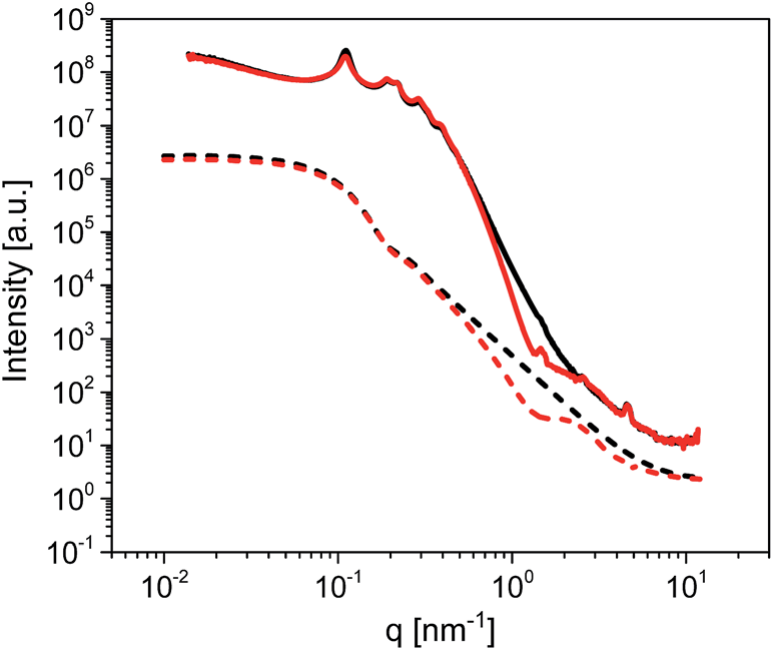
SAXS patterns of unmodified (black solid line) and ODPA-modified (red solid line) empty AAO membranes having a pore size of 38 nm. Dashed lines represent simulations based on a 2D core–shell cylinder model for unmodified (black dashed line) and modified (red dashed line) membranes. The simulations were performed for a core–shell cylinder, looking on-axis, with the following parameters: core diameter: 15 nm, polydispersity: 0.3 (in 45 points), shell thickness: 2.2 nm, polydispersity: 0.25 (in 45 points), cylinder angle phi: 0 (around-axis rotation), cylinder angle theta: 0 (face-on view), length: 600 nm (only affects the intensity) and background: 1 × 10^−1^ (avoiding deep dips). See also the ESI.†

The long-range ordering of both the unmodified and modified membranes was studied by Small Angle X-ray Scattering (SAXS) in order to estimate the thickness of the ODPA coating. Fig. 5 shows the measured SAXS patterns and corresponding simulations. The simulations are of 2D core–shell cylinders from the SasModels library.^24^ Similar models are applied elsewhere.^25,26^ The scattering pattern simulations are done with the cylindrical axis parallel to the beam, and using the scattering length densities estimated for bulk ODPA and alumina phases. Simulation trials with different shell parameters (cylinder diameter, shell thickness, and polydispersity) were carried out. The simulation with a shell thickness parameter of 2.2 nm closely approximates the SAXS pattern of the ODPA modified membranes.^27^ Hence, it is concluded that the ODPA coating has a thickness of *ca.* 2.2 ± 0.2 nm. The details of the SAXS measurements and some additional results are given in the ESI.†

### Sample preparation

A reproducible pore filling procedure was developed to embed HAT6 into nanochannels.^8^ The confined samples were prepared as outlined in ref. 8. In short, the membranes were outgassed in a vacuum of 10^−4^ mbar at 473 K for 12 hours, to clean the pores and remove adsorbed water. Then the membranes were transferred under vacuum to an argon-filled glovebox. The amount of material required to fill the membranes completely was calculated from the porosity and the volume of the membranes according to:1
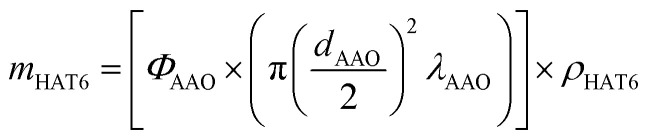
where *Φ*_AAO_ is the porosity, *d*_AAO_ is the diameter of the disk-shaped membrane, *λ*_AAO_ is the thickness of the membrane and *ρ*_HAT6_ is the bulk density of HAT6, found to be 0.92 g cm^−3^.^28^ The bulk-like density was assumed when HAT6 is confined into nanopores. The calculated amount of the liquid crystal and a small surplus was place on the top of the membrane and heated to 418 K in the isotropic state. At this temperature, the pores were filled by melt infiltration under an argon atmosphere for 48 h. After filling, the excess of the material on the top and bottom of the membranes was carefully scratched off with a sharp scalpel.

The filling degree of the membranes with HAT6 was estimated by Thermogravimetric Analysis (TGA). A complete pore filling was obtained for all the samples investigated in this study (see the ESI†).

### Differential scanning calorimetry (DSC)

DSC measurements were carried out using a Perkin Elmer DSC 8500 device. The sample (*ca.* 6–10 mg) was encapsulated in a standard 50 μl aluminum pan and measured in the temperature range from 173 K to 423 K with a heating/cooling rate of 10 K min^−1^. Nitrogen was used as a purge gas at a flow rate of 20 ml min^−1^. A baseline measurement was conducted by measuring an empty 50 μl aluminum pan under the same conditions. The obtained baseline was subtracted from the data measured for the sample. The second heating and the cooling runs were used to determine the phase transition temperatures and enthalpies. Moreover, the calibration of the machine was checked using an indium standard.

### Dielectric spectroscopy (DS)

The dielectric properties of the samples were measured by using a high-resolution ALPHA analyzer (Novocontrol) connected to a sample holder with an active head. The temperature of the sample was controlled by using a Quatro Novocontrol® temperature controller with nitrogen as a heating agent providing a temperature stability better than 0.1 K.

The measurements of HAT6 confined into nanochannels of the membranes were performed in a parallel plate geometry. This means that the disk-shaped samples were placed between two gold-plated brass electrodes with a diameter of 10 mm or 15 mm depending on the outer diameter of the membranes. Spacing between electrodes was defined by the thickness of the membranes. Bulk measurements were conducted using a commercially available liquid crystalline cell. The liquid crystalline cell, having square (10 mm × 10 mm) patterned ITO-coated electrodes and an average cell gap of 4 μm, was purchased from Instec, Inc. (Colorado, USA). It was capillary filled with HAT6 in the isotropic phase at 383 K by placing a small amount of sample at the front gate of the cell.

The complex dielectric permittivity ε*(*f*) = *ε*′(*f*) − i*ε*′′(*f*) was measured by temperature scans with a heating and cooling rate of 1 K min^−1^ at a constant frequency of 35 kHz. Here 
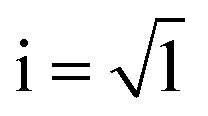
 symbolizes the imaginary unit and *f* denotes the frequency where *ε*′ and *ε*′′ are the real and imaginary (loss) parts of the complex dielectric permittivity. More details about BDS can be found in ref. 29.

Three heating/cooling cycles, in the temperature range from 340 K to 383 K for smaller pore sizes (*d* < 34 nm) and that from 350 K to 383 K for the larger pore sizes (*d* > 34 nm), were employed to probe all samples. Similarly, three heating/cooling cycles were applied for HAT6 in the liquid crystalline cell in the temperature range from 350 K to 383 K.

### Polarizing optical microscopy (POM)

The liquid crystalline texture and the alignment of the columns were investigated by POM for bulk HAT6. The measurements were carried out by using a Zeiss Axioskop Scope A1 optical microscope, with crossed polarizers, connected to a Linkam THMS600 heating stage. The stage was equipped with a liquid nitrogen Dewar allowing a precise control of heating and cooling rates.

The ITO-coated liquid crystalline cell used in the DS measurement was also employed for the POM investigations. The temperature program applied for the DS measurements was also used for the POM measurements.

## Results and discussion

### Mesomorphic properties of bulk

**Fig. 6:**
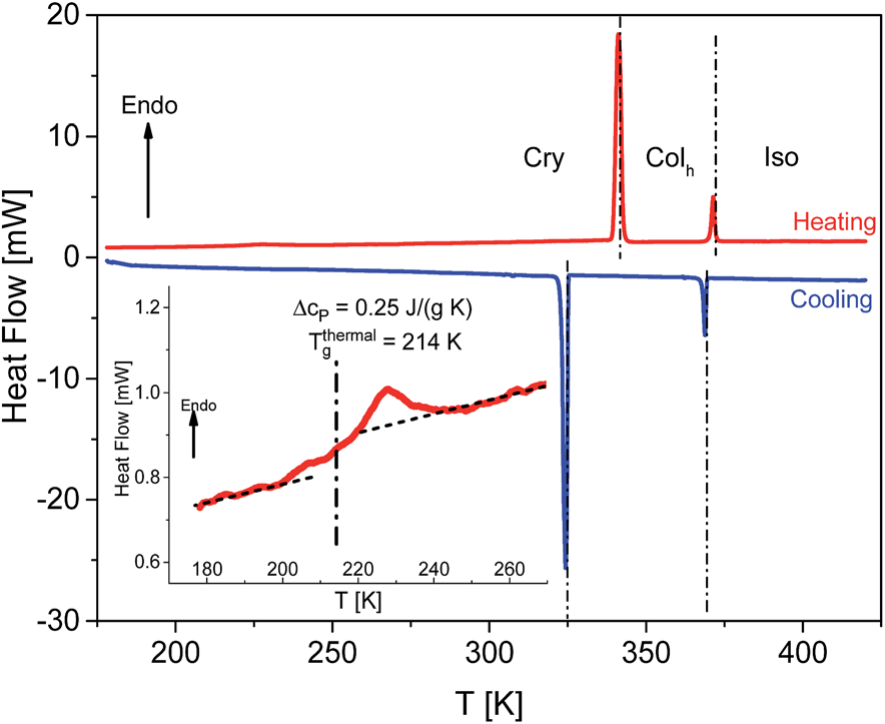
DSC thermograms of HAT6 during heating (red line) and cooling (blue line) with a heating/cooling rate of 10 K min^−1^. Dashed lines point out the phase transition temperatures upon heating and cooling. The inset enlarges the temperature range between 170 K and 270 K for the heating run.

The mesomorphic properties of HAT6 in the bulk state were studied by DSC and POM. DSC thermograms of HAT6 are given for the second heating and cooling runs in Fig. 6. The phase transition temperatures and enthalpies are estimated from the maximum positions of the peaks and the area under the peaks respectively and given in Table 2. Moreover, the Col_h_–Iso phase transition temperatures were also determined by DS from the first derivative of *ε*′ with respect to temperature. A hysteresis between the cooling and heating cycles was observed where the hysteresis is more pronounced for Cry–Col_h_ transition (Δ*T* = 15.9 K) than Col_h_–Iso transition (Δ*T* = 2.8 K).

**Table 2:** Phase transition temperatures and enthalpies determined together with the literature values

Source	Run	Phases, transition temperatures and enthalpies	
		DSC	DS
In this study	Cooling	Cry, 325.9 K (52.5 J g^−1^); Col_h_, 369.3 K (6.8 J g^−1^); Iso	Col_h_, 369.6 K; Iso
	Heating	Cry, 341.8 K (51.3 J g^−1^); Col_h_, 372.1 K (6.8 J g^−1^); Iso	Col_h_, 370.3 K; Iso
Ref. 30	Heating	Cry, 340.6 K (50.6 J g^−1^); Col_h_, 371.5 K (5.9 J g^−1^); Iso	
Ref. 19	Heating	Cry, 342.7 K; Col_h_, 372.7 K; Iso	
Ref. 31	Heating	Cry, 342.0 K (49.9 J g^−1^); Col_h_, 372.3 K (6.8 J g^−1^); Iso	
Ref. 8	Heating	Cry, 342.0 K (49.9 J g^−1^); Col_h_, 372.3 K (6.8 J g^−1^); Iso	

A step indicating a thermal glass transition was observed in the temperature range between 170 K and 270 K, see the inset of Fig. 6. The mid-step position of the step was taken to determine the thermal glass transition temperature (*T*^thermal^_g_) of 214 K. The change in the specific heat capacity Δ*c*_p_ was found to be 0.25 J K^−1^ g^−1^. Undergoing a glass transition implies that there are some disordered or amorphous structures within the material. Such disorder, which leads to a glass transition, may be caused by a nanophase separation of the alkyl chains and the aromatic core of HAT6, which is evidenced by the amorphous halo observed in the X-ray pattern of HAT6.^31^ Similarly, Yildirim *et al.*^30^ reported a glass transition for HAT6 also detected by DSC. In contrast to our findings, they found a *T*^thermal^_g_ of 186 K. Furthermore, Krause *et al.*^31^ observed a step, which might indicate a glass transition, in the temperature range of 180–220 K upon cooling. They did not observe a glass transition during the heating run. Clarifying these contradicting findings requires further detailed calorimetric investigations, such as conventional DSC investigations covering temperatures lower than 150 K or Hyper/Flash DSC investigations allowing for higher heating rates.

**Fig. 7:**
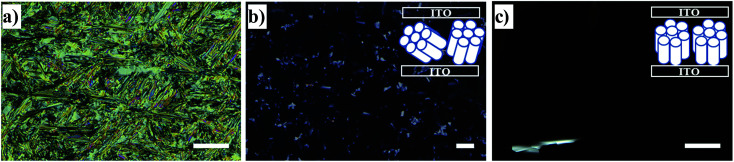
POM images of HAT6, recorded under crossed polarizers, in the liquid crystalline cell having ITO-coated electrodes (a) at 328 K in the Cry phase during the 1^st^ heating run from room temperature, (b) at 363 K in the Col_h_ phase during the first heating run and (c) at 363 K in the Col_h_ phase during the third heating run. The insets of (b) and (c) are the drawings representing the molecular ordering for a given image. Scale bars represent 200 μm.

Fig. 7 illustrates the texture and the column alignment of bulk HAT6 obtained by POM. Upon first heating from room temperature, in the Cry phase at 328 K the texture is very bright under cross polarizers. This indicates a lack of a homeotropic alignment due to tilted columns in the Cry phase (herringbone-like crystal packing^32,33^) causing a high birefringence. This results in a bright texture with colored crystal domains.^4^ A fan-shaped texture accompanied by dark areas was observed during the first heating ramp in the Col_h_ phase at 363 K. The fan-shaped texture is typical of a Col_h_ phase of DLCs. It indicates that a column alignment in large spatial regions is not present. As shown in the inset of Fig. 7b, the columns are randomly tilted.^6,34^ In addition to the fans, small dark areas were also observed, which indicates that the columns are partially aligned homeotropically among the tilted columns causing a minimal birefringence. However, during the third heating run (after applying two heating/cooling cycles in the range from 350 K to 383 K) at 363 K, large dark areas are observed (see Fig. 7c), where the columns are mostly aligned homeotropically. The reason for the homeotropic alignment of the columns in large spatial regions compared to the first heating probably is the temperature program applied due to the self-healing ability of DLCs for the structural defects with thermal annealing. It could also be reasoned that the heating/cooling rate applied, 1 K min^−1^, is not slow enough to align the columns during the first heating due to what is assumed to be slow orientation kinetics.

### Phase behavior under confinement

**Fig. 8:**
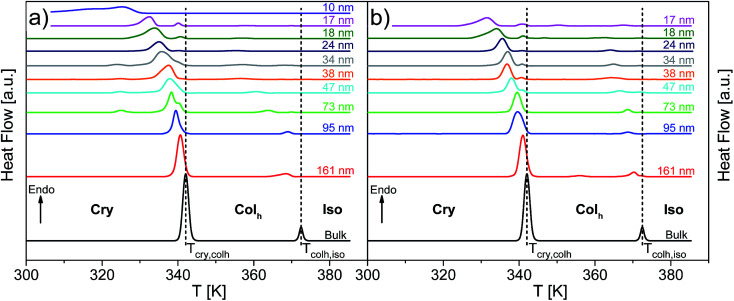
DSC thermograms of bulk HAT6 and HAT6 confined into (a) unmodified and (b) ODPA-modified nanopores of the membranes having different pore sizes as indicated (second heating cycle). The dashed lines indicate the phase transition temperatures of bulk HAT6. The curves are shifted along the *y*-scale for sake of clarity.

Fig. 8 depicts the DSC thermograms of bulk HAT6 as well as HAT6 confined into the nanopores of unmodified and ODPA-modified AAO membranes. It is concluded that the confined HAT6 undergoes both phase transitions (Cry–Col_h_ and Col_h_–Iso) with decreasing pore size for both unmodified and modified pore walls. One exception to this is observed with HAT6 confined within 12 nm pores in a silicon membrane, where the Col_h_–Iso phase transition is completely suppressed. Such a complete suppression of the phase transition has previously been reported for rod-like LCs.^22^

**Fig. 9:**
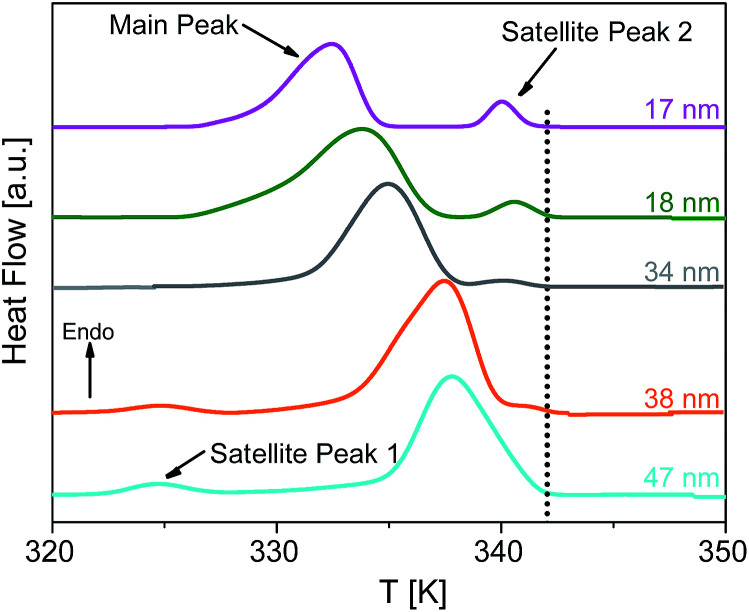
DSC thermograms of HAT6 confined into unmodified nanopores of the membranes having different pore sizes as indicated, enlarging the temperature range from 320 K to 350 K for the heat flows of the samples given in Fig. 8. The dotted line indicates the Cry–Col_h_ phase transition temperature of the bulk. The curves are shifted along the *y*-scale for sake of clarity.

As previously reported for the phase behavior of HAT6 under confinement for a limited range of pore sizes,^8^ three conclusions can be drawn for both kinds of samples with unmodified and modified pore walls. Firstly, both phase transition temperatures shift to lower temperatures with decreasing pore size. Secondly, both phase transition peaks split into two or three peaks for pore sizes smaller than 95 nm (see Fig. 9). The appearance of the so-called satellite peaks in addition to the main transition peak for the smaller pore sizes was reported for the nanoconfined pyrene-based DLC and also for HAT6.^8,9^ In a first interpretation, the satellite peaks can be due to remaining bulk-like HAT6 located at the surface of the sample although attempts were made to remove it carefully. A similar interpretation is used elsewhere.^35^ Secondly, the main and the satellite peaks might be assigned to different configurations of the molecules near the pore walls and the pore center. This conclusion is supported by the observation that the satellite peaks are shifted to lower temperatures with respect to the phase transition of the bulk and depend slightly on pore size. For these reasons the second interpretation is favored. However, such assignments can prove to be controversial due to the lack of experimental techniques available to characterize the different structures and their location within the pore space. Thirdly, for both phase transitions the enthalpies decrease with decreasing pore size. However, this conclusion cannot be drawn directly from Fig. 8, as the measured values are normalized to the confined mass of HAT6. This third conclusion is discussed in detail below.

**Fig. 10:**
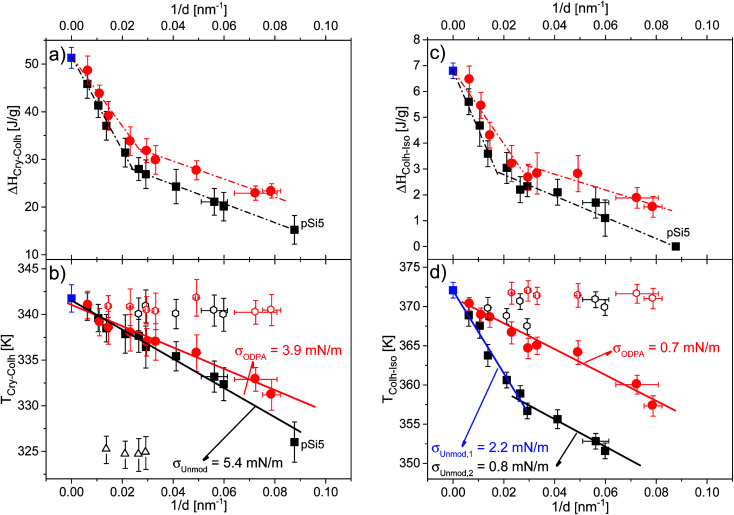
Dependencies of the phase transition enthalpies for (a) the Cry–Col_h_ transition and (c) the Col_h_–Iso transition, as well as the dependencies of the phase transition temperatures for (b) the Cry–Col_h_ transition and (d) the Col_h_–Iso transition *versus* inverse pore size. Blue squares indicate data for bulk HAT6, black symbols indicate data for HAT6 confined into unmodified membranes and red symbols indicate data for HAT6 confined into ODPA-modified membranes (filled circles: main peak; open symbols: satellite peak). Dashed-dotted lines are a guide for the eye. Solid lines are the fits of eqn (2) to the dependencies. Note that for the samples with modified pore surfaces, the pore diameter was corrected regarding the estimated thickness of the surface layer.

The dependence of phase transition enthalpies normalized to sample mass inside the pores and the phase transition temperatures are shown in Fig. 10. Moreover, the effect of the different host/guest interactions on the phase behavior under nanoconfinement is revealed by comparing the behavior of HAT6 confined in both unmodified and ODPA-modified pores. The ODPA modification forms a stable grafted alkyl chain monolayer, which results in hydrophobic pore walls with a lower value of the surface energy compared to unmodified pores.^36,37^ The thickness of the ODPA coating was reported to be 1.8–2.4 nm on the aluminum oxide surface,^38^ which is comparable to the thickness revealed from SAXS investigations. Hence, the modification leads to an observed decrease of the pore diameter of *ca.* 4.4 nm for the ODPA-modified AAO membranes. The influence of the decreased pore size should be greater for smaller pores than for larger ones considering inverse pore sizes. Therefore, the dependencies given in Fig. 10 for the sample with the ODPA-modified pore surfaces were drawn considering a 4.4 nm decreased pore size. However, the thickness of the ODPA coating is not known for the pores filled with HAT6. In the ESI, Fig. S4† shows the dependencies for the nominal pore sizes. When the modified pores are filled with HAT6, the thickness of the coating can, in principle, have a value between 0 nm and 4.4 nm, whereas in reality values tend to lie towards the latter end of this scale.

The dependencies of the phase transition enthalpies of the main peak, normalized to the mass of the confined material, *versus* inverse pore size are given in Fig. 10a and c for the Cry–Col_h_ and the Col_h_–Iso transitions respectively. The normalized phase transition enthalpies decrease with decreasing pore size, which provides evidence that a portion of confined HAT6 does not undergo the phase transition. This part of the confined HAT6 should be disordered and may have an amorphous structure. As discussed in ref. 8 and 9, the observed increase in the surface curvature, with decreasing pore size, leads to stronger elastic distortion of the ordered phase, which prevents the molecules from forming an ordered structure and consequently limits the amount of observed ordered phase.

The dependencies of the enthalpies for the Col_h_–Iso transition reveal that more than half of the material confined in the pore (by volume) is disordered for pore sizes smaller than 47 nm. This is an amount that cannot be neglected in the interpretation of the results for nanoconfined HAT6. The spatial location of this disordered portion inside the pore cannot be assigned by methods characterizing the structure such as X-ray diffraction or neutron scattering. As it has been discussed that the disordered portion is likely located at the center of the pore due to the divergence of the excess energies toward the pore center.^7,13,15^ A disordered core at the center of the pore was also visualized by molecular dynamics simulations of confined HAT6.^17^

The pore size dependencies of the phase transition enthalpies show stronger dependence on pore size for larger pores in comparison to smaller ones. Similar findings were reported for a series of organic materials confined within nanopores.^39^ The surface area of the pores per unit of mass is significantly higher for smaller pores. This can lead to stronger confinement effects on the enthalpies for smaller pores for nanoconfined materials, which could explain similar trends observed with different nanoconfined materials. In addition, higher values of the transition enthalpies were found for surface modified samples compared to unmodified ones. This means that the amount of ordered HAT6 increases for the samples with the ODPA-modified pore walls even though there is still a considerable amount of disordered material inside the pores, most likely located in the pore center.

Fig. 10b and d show the dependencies of the phase transition temperatures on inverse pore size for both phase transitions. It can be seen that the phase transition temperatures decrease with decreasing pore size. In general, the pore size dependence of phase transition temperatures can be well described by the Gibbs–Thomson equation for a variety of materials including liquid crystals.^8,9,40,41^ The Gibbs–Thomson equation reads:2
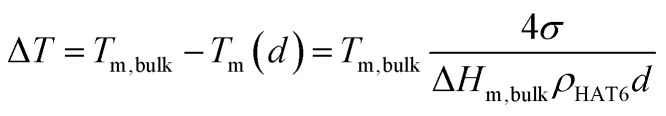
where *T*_m,bulk_ is the phase transition temperature of the bulk material, *d* is the pore diameter, *T*_m_(d) is the phase transition temperature of HAT6 within the pores of diameter, *d*, and *σ* is the surface tension of the interface. Δ*H*_m,bulk_ is the phase transition enthalpy of the bulk material.

For the phase transition from the plastic crystalline to the hexagonal ordered phase the data seem to follow the Gibbs–Thomson equation for both types of samples probed. The surface tensions were calculated to be 5.4 mN m^−1^ and 3.9 mN m^−1^ from the dependencies of Cry–Col_h_ phase transition temperatures for the samples with unmodified and ODPA-modified pore walls, respectively. The ODPA modification makes the pore wall more hydrophobic in comparison to the unmodified pore walls. Therefore, the interfacial tension is lower for the samples with modified pore walls than for unmodified ones. Fig. 10d depicts that the dependence of phase transition temperatures of the Col_h_–Iso phase transition changes at a pore size of *ca.* 38 nm for the samples with the unmodified pore walls. Assuming that both pore size dependencies can be described by a Gibbs–Thomson equation for pore sizes larger than 38 nm, the surface tension was calculated to be 2.2 mN m^−1^, whilst for pore sizes smaller than 38 nm a value of 0.8 mN m^−1^ was calculated. As it will be discussed below in more detail this change in the pore size dependence of the phase transition temperature for the Col_h_–Iso phase transition goes along with a change in the dominating order. It is also important to note that the pore size dependence of the Col_h_–Iso phase transition observed here is different from that reported in ref. 13. The reason for this is not quite clear and requires additional experiments.

The pore size dependency of the phase transition temperature of the Col_h_–Iso phase transition for the samples with the modified pore walls can be described with a single Gibbs–Thomson equation where a value of 0.7 mN m^−1^ was calculated for the surface tension. This value is quite similar to the one obtained for the unmodified pore walls for smaller pore sizes. It might be concluded that the type of dominating anchoring is the same in both cases (see Table 3). Moreover, a study on the effect of orientation on the reduction of the phase transition temperature revealed by specific heat spectroscopy for a confined rod-like liquid crystal^42^ demonstrated the stronger decrease of the transition temperatures for a radial configuration. In addition, the favorable interactions between the aromatic core and the polar surface of the unmodified membranes enforce likely face-on anchoring leading to homeotropic radial configuration (logpile). Hence, it can be concluded that the homeotropic radial configuration is the dominant type of ordering found in larger unmodified pores. Recently, Zhang *et al.*^19^ also found a dominant logpile configuration for HAT6 confined in native nanopores of AAO membranes.

**Table 3:** The dominating ordering of HAT6 molecules inside the nanopores revealed by means of different techniques. HAT6 confined into the silicon membrane (12 nm) is not presented in the table since the Col_h_–Iso phase transition is completely suppressed for this sample

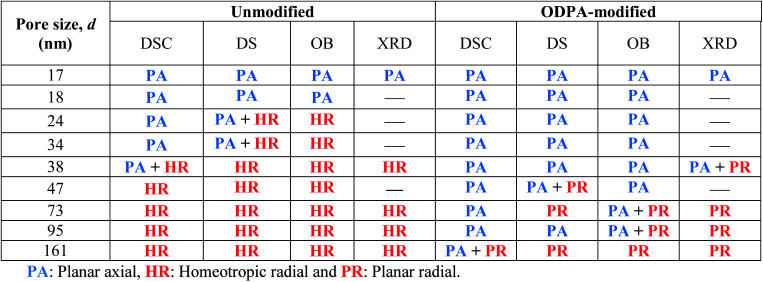

For the smaller unmodified pores and for the modified pores, it was assumed that the dominating type of ordering is a planar axial configuration. The similar values obtained for the surface tensions (*σ*_Unmod,2_ ≈ *σ*_ODPA_, Fig. 10d) further support this assumption. The planar configuration is assumed for samples with the modified pore walls due to the similarity between the alkyl chains and the alkyl chains grafted to the surface of the modified membranes. In contrast, findings in the literature for confined HAT6 point out logpile configurations in unmodified small pores^19^ and circular concentric (planar radial) configurations in nanopores grafted by alkyl chains.^18^ Although the calorimetric investigations give a clear picture of the phase behavior under confinement, they provide only a limited understanding to predict the dominating configuration in the nanopores based on the pore size dependencies of the phase transition temperatures and enthalpies. These predictions will be reevaluated with respect to the orientational order characterized by DS in the next section.

### Collective orientational order

**Fig. 11:**
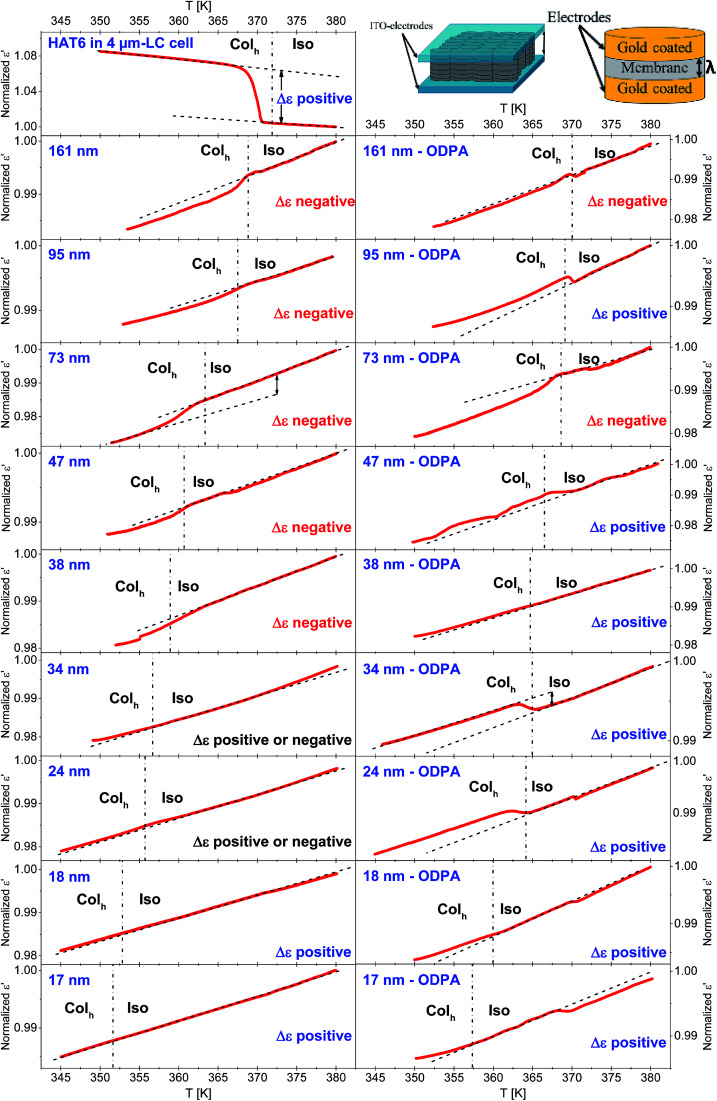
Normalized dielectric permittivities as a function of temperature during the third heating run for the bulk, and samples with unmodified and ODPA-modified pore walls as indicated. All measurements were done with a heating/cooling rate of 1 K min^−1^ at a frequency of 35 kHz. The dashed-dotted lines indicate the Col_h_–Iso phase transition temperatures determined by DSC. The dashed lines represent the temperature dependencies of *ε*′ extrapolated from Col_h_ and Iso phases. The insets on the right-top are the drawings illustrating the measurement geometry used for the dielectric measurement for bulk HAT6 in the cell and the confined HAT6.

The collective orientational order of HAT6 in the bulk and confined HAT6 was revealed by dielectric spectroscopy. Of course, dielectric spectroscopy is not a tool to estimate the structure directly. Dielectric spectroscopy is sensitive to dipole orientation (dipole vector with respect to the outer electrical field). Therefore, a change in the orientation at the phase transition can be monitored when a change of the orientation of the dipole moment is involved. Together with a reference measurement on the bulk material where the orientation is known a conclusion based on facts about the orientation can be drawn. The measurements were carried out at a frequency of 35 kHz, where results from the third heating run are provided in Fig. 11. A frequency of 35 kHz was selected for these investigations as no dielectric active processes due to molecular fluctuations for HAT6 take place in the temperature range from 350 K to 383 K. In this temperature range relaxation processes take place at frequencies between 1.9 MHz and 1.2 GHz (obtained from the extrapolation of the data given in ref. 30). Hence, a measurement frequency of 35 kHz is significantly lower than the frequency window of the dielectric relaxation processes, and the measurements shown in Fig. 11 correspond to quasi-static dielectric measurements where only the collective orientation of HAT6 contributes to *ε*′, which can be considered as frequency-independent.

In the DS measurements, the cylindrical pores were oriented parallel to the electric field applied due to the parallel plate geometry (see the inset of Fig. 11). Therefore, the system can be considered as AAO and HAT6 capacitors connected in parallel yielding an additive response.^22^ Thus, the absolute value of the permittivity of such a system is related to the porosity of the membrane. For this reason, the measured *ε*′ values were normalized with respect to the values of *ε*′ at 383 K for each sample.

The excess permittivity (Δ*ε*) can be defined as the difference between the temperature dependence of *ε*′ in the Col_h_ phase, and that of *ε*′ extrapolated to the Col_h_ phase from the dependence in the Iso phase. A positive Δ*ε* was determined for bulk HAT6, where HAT6 molecules are homeotropically aligned, which was also confirmed by POM. The positive Δ*ε* indicates that the columns are perpendicularly aligned to the electrodes. Hence, a positive Δ*ε* can be attributed to a dominating axial configuration, whilst a negative Δ*ε* corresponds to a dominating radial configuration. In some cases, from the overview given in Fig. 11 it is hard to detect whether Δ*ε* is positive or negative. Therefore, enlarged figures are prepared and added to the ESI (see Fig. S6–S10).†

For the unmodified pores with diameters smaller than 38 nm, the Δ*ε* was estimated to be positive and assigned to a planar axial configuration. For pore sizes greater than 38 nm, a negative Δ*ε* was observed and assigned to the homeotropic radial configuration. This is in good agreement with the discussion given above concerning the pore size dependency of the phase transition temperatures. The DSC investigations indicate that a change in the anchoring type occurs at a pore diameter of *ca.* 38 nm, which was also observed using DS. Conversely, a positive value of Δ*ε* was found for samples with modified surfaces, expect for the pore sizes of 73 nm and 161 nm. This has been attributed to the planar axial configuration, where a planar radial configuration was assumed for 73 nm and 161 nm due to the negative value of Δ*ε*. For the sample with a pore size of 47 nm with modified pore walls, generally a positive value of Δ*ε* was found; however it was observed that the phase transition occurs in several steps (see also Fig. S5 in the ESI†). As shown recently with high resolution optical birefringence experiments on HAT6 confined within porous silica and molecular dynamic simulations,^17^ such steps can be attributed to a circular concentric (planar radial) configuration. Therefore, it might be concluded that the orientation of HAT6 in 47 nm modified AAO pores also possesses a circular concentric (planar radial) configuration.

In addition to the dielectric investigations the molecular ordering for the samples presented here was also characterized by optical birefringence (OB) measurement and temperature dependent X-ray diffraction (XRD). These measurements will be published elsewhere^43^ because they would increase the length of this publication too much. Nevertheless, the results of these measurements are included here in Table 3. Some preliminary results have been already published discussing also the methodology of the measurements (see the ESI of ref. 17). Combining the results of the interpretations based on the DSC investigation and the ordering characterized by DS, OB and XRD, a general picture of the molecular ordering inside the unmodified and ODPA-modified nanopores was obtained (see Table 3). In most cases the results obtained from the DSC and dielectric measurements agree with the data obtained with OB and XRD. A minor difference between DS and OB findings might be caused by the small differences in the sample preparation. However, it is argued in ref. 15 that the dipolar orientation of the polar ether groups of alkyl chains is sensed by DS, whereas the orientation of the aromatic cores is observed by OB. This may explain the small differences observed in the determined configurations by DS and OB.

According to the above discussions and Table 3, the picture of the molecular ordering of HAT6 inside the unmodified and ODPA-modified nanochannels of AAO membranes can be concluded as follows: a model representing the molecular ordering of HAT6 inside nanopores should include three idealized major layers: a disordered layer probably located at the center of the pore, an axial ordered layer near the center and a radial ordered layer near the pore walls. For HAT6 in the unmodified nanopores, a transition of the dominant configuration from planar axial (Fig. 1d) to homeotropic radial (Fig. 1c) configuration was detected in the pore size range from 24 nm to 38 nm. On the other hand, for HAT6 in the ODPA-modified nanopores the transition from planar axial (Fig. 1f) to planar radial (Fig. 1e) configuration was observed in the pore size range from 73 nm to 95 nm.

## Conclusions

The influence of cylindrical nanoconfinement on the phase behavior and molecular ordering – two main properties determining the applications of a DLC in nanotechnology – was explored by DSC and DS for HAT6 confined into the nanopores of AAO and silica membranes. Pore sizes from 161 nm down to 12 nm were explored. Moreover, the different host/guest interactions and their influence on the phase behavior as well as molecular ordering were studied by comparing their behavior and ordering in unmodified and ODPA-modified pores. Prior to the investigations, the membranes and the confined samples were well characterized by means of volumetric N_2_-sorption, scanning electron microscopy, FTIR, SAXS and TGA.

The pore size dependencies of the phase transition enthalpies and temperatures were obtained by DSC. It was observed that the phase transition enthalpies decrease with decreasing pore size, which indicates that an increasing fraction of HAT6 does not undergo any phase transitions, and it is thought that this fraction is probably located in the center of the pore. Moreover, the phase transition temperatures decrease with decreasing pore size. These pore size dependencies of the phase transition temperatures were described by the Gibbs–Thomson equation for both transitions. For the Cry–Col_h_ transition, a lower interfacial tension was found for the samples with modified pore walls in comparison to the samples with unmodified pore walls. For the pore size dependency of the Col_h_–Iso transition temperatures for the samples with unmodified pore walls, a change from a stronger to a weaker dependency was observed with a pore size of around 38 nm. Such a change implies that there is an alteration in the dominating order. Therefore, by considering the host–guest interaction it can be concluded that the dominating type of ordering is a homeotropic radial configuration with larger pores (*d* > 38 nm) and a planar axial configuration for smaller pores (*d* < 38 nm). However, the dependencies of the Col_h_–Iso phase transition temperatures for the samples with modified pore walls were approximated by using only one Gibbs–Thomson equation. Similar values for the surface tension for the samples with modified pore walls to those estimated for samples with unmodified pore walls at lower pore sizes (*σ*_Unmod,2_ ≈ *σ*_ODPA_) were found. Hence, it is concluded that for the samples with modified pore walls the dominating type of ordering is also the planar axial configuration.

The collective orientational order of nanoconfined HAT6, corresponding to the dominating ordering in the pore, was probed by DS at a constant frequency of 35 kHz. Similar to the DSC findings, DS investigations revealed that for the unmodified pores a homeotropic radial orientation for the larger pores (*d* > 38 nm) and a planar axial orientation for the smaller pores (*d* < 38 nm) were found as dominating forms of ordering. For ODPA-modified pore walls, the planar axial configuration is assigned as the dominating type expect for the pore sizes of 73 nm and 161 nm. Moreover, OB and XRD studies on the samples discussed here mostly agreed the molecular orderings determined by DS.

In summary, we have reported ODPA surface modification as a promising strategy for controlling the molecular ordering of DLCs inside the nanopores of metal oxide membranes. Our results indicate that the dominating planar axial configuration, which is the only configuration suitable for electronic applications, was successfully achieved by ODPA modification for most of the pore sizes probed. Moreover, the higher phase transition enthalpies and temperatures observed for the samples with modified pore walls compared to the unmodified ones imply a significant improvement in the amount of ordered HAT6 present within the pores.

## Conflicts of interest

The authors declare no competing financial interest.

## Supplementary Material

NA-001-C8NA00308D-s001
