# Supplementary material for: Collective orientational order and phase behavior of a discotic liquid crystal under nanoscale confinement
Source: Nanoscale Adv. 2018 Dec 3;1(3):1104–16. doi: 10.1039/c8na00308d (PMC9473266; doi:10.1039/c8na00308d)
Supplement: NA-001-C8NA00308D-s001 [file NA-001-C8NA00308D-s001.pdf]

Electronic Supplementary Material (ESI) for Nanoscale Advances.
This journal is © The Royal Society of Chemistry 2018

Electronic Supplementary Information

Collective Orientational Order and Phase Behavior of a DLC Under Nanoscale Confinement

Arda Yildirim[1], Kathrin Sentker[2], Glen Jacob Smales[1], Brian Richard Pauw[1], Patrick Huber[2], and Andreas Schönhals[1]

[1]Bundesanstalt für Materialforschung und -prüfung (BAM), Unter den Eichen 87, 12205 Berlin, Germany

[2]Institut für Materialphysik und -technologie, Technische Universität Hamburg, Eißendorfer Str. 42, 21073 Hamburg, Germany

Attenuated Total Reflection Fourier Transform Infrared Spectroscopy (ATR-FTIR)

ATR-FTIR was employed to confirm the surface modification of the membranes. The measurements of empty unmodified and modified membranes were carried out at room temperature using a Thermo Scientific Nicolet 6700 FTIR with a Smart Orbit diamond ATR accessory. The number of accumulated scans was 32 with a resolution of 4 cm^{-1} and the wavenumber range of 4000-400 cm^{-1}.

Peaks for the alky C-H stretching should appear for the FTIR spectra of the modified membranes, and its appearance can be used as an indication to confirm the ODPA-modification. Figure S1 shows that the peaks at 2850 cm^{-1} and 2930 cm^{-1}, corresponding to symmetric and antisymmetric alky C-H stretching respectively appear for the modified empty membranes (red and blue lines). As it is expected, these peaks were not observed in FTIR spectra for the unmodified membranes (black and green lines).

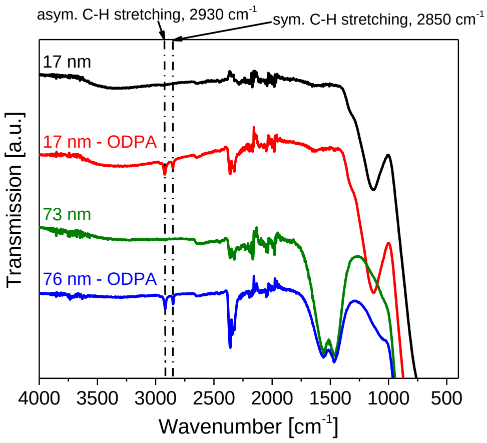

Figure S1. FTIR spectra of the ODPA-modified and unmodified aluminum oxide membranes as indicated.

Small Angle X-ray Scattering (SAXS)

Long-range ordering was studied by small angle X-ray scattering (SAXS) in the "MAUS": a heavily customized Xeuss 2.0 (Xenocs, France). X-rays are generated from a microfocus X-ray tube with a copper target, followed by a multilayer optic to parallelize and monochromatize the X-ray beam to a wavelength of 0.154 nm. The detector consists of an in-vacuum motorized Eiger 1M, for this investigation placed at distances of 208, 558, and 1258 mm from the sample. After correction, the data from the different distances are combined into a single curve. The space between the start of the collimation until the detector is a continuous, uninterrupted vacuum to reduce background. The membranes (discs) were mounted with their surface perpendicular to the beam in the evacuated sample chamber. The resulting data has been processed using the DAWN software package[1,2] with the following processing steps in order: masking, correction for counting time, dark-current, transmission, primary beam flux, background (no sample in the beam), flat-field, polarization and solid angle, followed by azimuthal averaging. The data has not been scaled to absolute units. Photon counting uncertainties were estimated from the raw image, and propagated through the correction steps. For the alignment experiment, the membrane was held upright between two LEGO bricks, on top of a PhysikInstrumente H811.I2V hexapod. 200 second exposures were taken at various tilt angles, to seek the tilt angle that would produce a radially isotropic pattern.

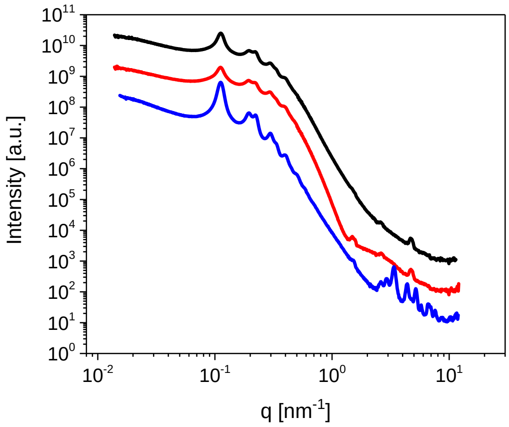

Figure S2. SAXS patterns of unmodified (black) and ODPA-modified (red) AAO membranes with 38 nm diameter pores as well as that of HAT6 confined AAO membranes with 38 nm pore size (blue). The curves are shifted along the y-scale for clarity.

Figure S2 shows the SAXS patterns of the samples. It is concluded that the pores are 100% filled for HAT6 confined into AAO with 38 nm pore diamater.

Thermogravimetric Analysis (TGA)

The filling degree of the membranes with HAT6 was controlled by TGA. TGA measurements were carried out by a Seiko TG/DTA 220 with a heating rate of 10 K/min, from room 303 K to 1000 K, under synthetic air to burn all the organic compound confined into the nanopores.

Figure S3 shows TGA curves for bulk HAT6, the confined HAT6 in unmodified and ODPA-modified AAO membranes as well as the modified empty membrane. All the samples shown in Figure S3 are thermally stable at least up to 550K, which ensures that there was no thermal degradation during the sample preparation and the measurements.

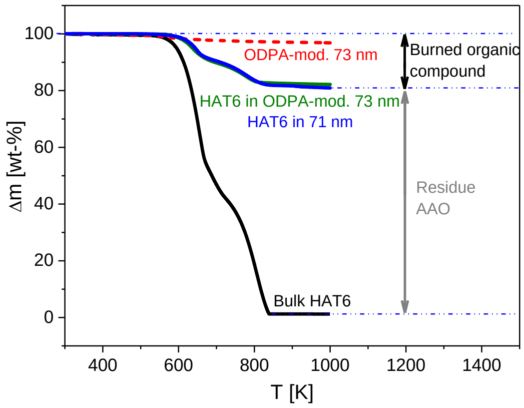

Figure S3. TGA curves for HAT6 in the bulk (black solid line), HAT6 confined into unmodified AAO membrane (blue solid line), HAT6 confined into ODPA-modified AAO membrane (green solid line) and empty ODPA-modified AAO membranes (red dashed line). TGA curves given in here for the membranes having the pore size of 73 nm.

From the estimated masses, the degree of filling can be estimated. Complete pore filling was obtained for all the samples shown in this study.

<u>Phase Behavior Under Confinement by DSC</u>

ODPA modifications of the nanopores causes ca. 4.4 nm decrease in the diameter of the pores for the empty membranes. Nevertheless, thickness of the ODPA-modification could not be measured for the HAT6 filled coated pores. Therefore, Figure S4 shows the dependencies of the phase transitions temperatures and enthalpies on inverse pore size without considering the narrowing of the pore size for ODPA-modified samples. Comparing the dependencies shown in Figure 10 and Figure S4, it is concluded that the pore narrowing has no considerable effect on our discussions based on these dependencies.

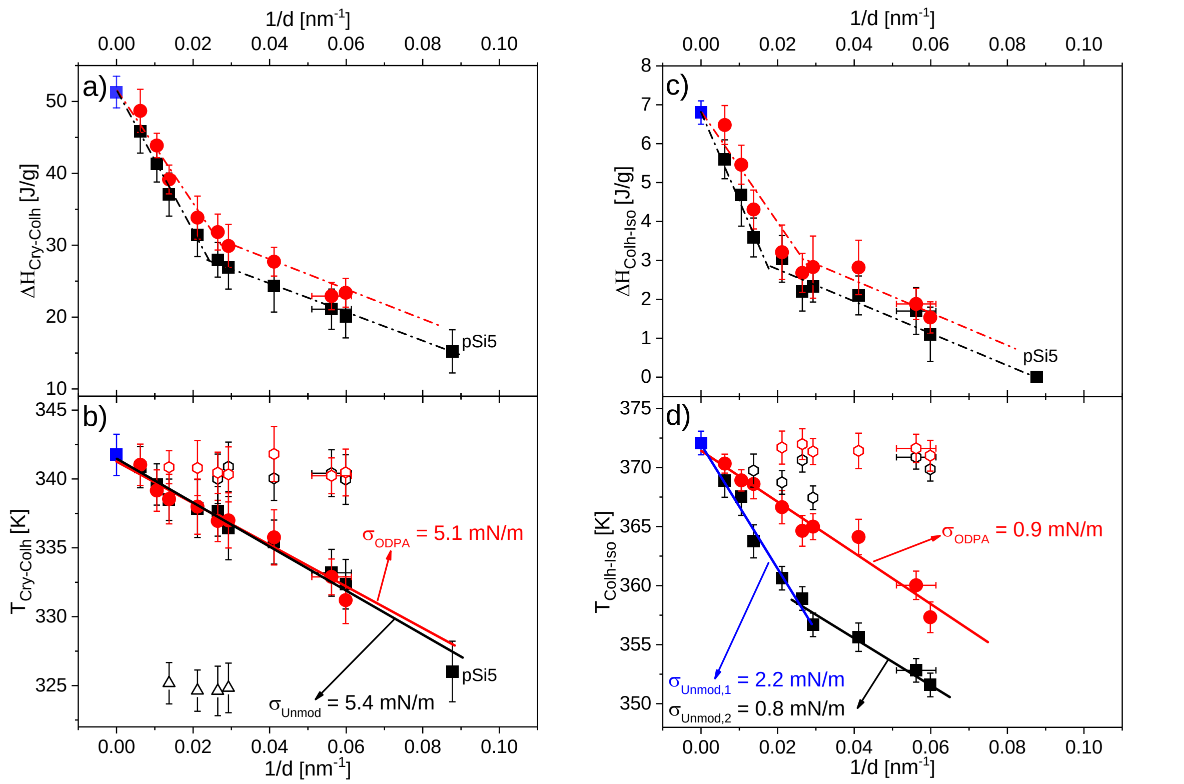

Figure S4. Inverse pore size dependencies of the phase transition enthalpies for (a) the Cry-Colh transition and (c) the Colh-Iso transition, as well as that of the phase transition temperatures for (b) the Cry-Colh transition and (d) the Colh-Iso transition. Blues squares indicate data for bulk HAT6, black symbols indicate data for HAT6 confined into unmodified membranes and red symbols indicate data for HAT6 confined into ODPA-modified membranes (filled circles: main peak, open symbols: satellite peak). Dashed-dotted lines are guide for eyes. Solid lines are the fits of eqn (2) to the dependencies.

Collective Orientational Order by DS

DS investigations showed unusually the phase transition takes place in several steps for HAT6 confined into the ODPA-modified nanopores of AAO membrane with a pore size of 47 nm. Therefore, the DS result for these samples is represented in Figure S5 in order to show the steps clearly.

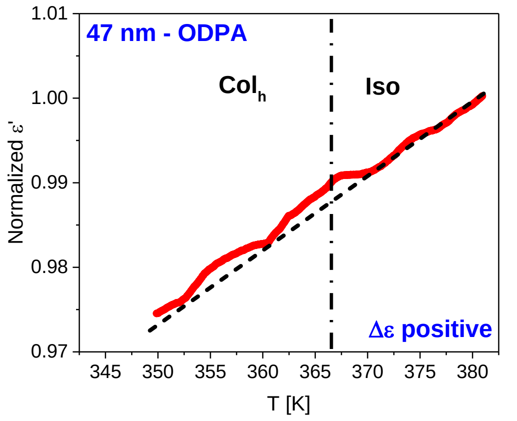

Figure S5. Normalized dielectric permitivities as a function of temperature during the third heating run for the sample with a pore size of 47 nm with modified pore walls. The dashed-dotted lines indicate the Col$_h$-Iso phase transition temperatures determined by DSC. The dashed lines represent temperature dependencies of ε′ extrapolated from Col$_h$ and Iso phases.

In some cases from the overview given in Figure 11 it is hard to detect whether Δε is positive or negative. Therefore, enlarged figures are prepared for some sample and given in Figure S6 -Figure S10.

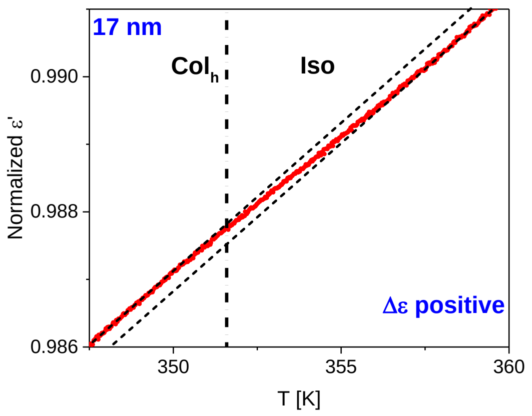

Figure S6. Normalized dielectric permitivities as a function of temperature during the third heating run for the sample with a pore size of 17 nm with unmodified pore walls. The dashed-dotted lines indicate the Col$_h$-Iso phase transition temperatures determined by DSC. The dashed lines represent temperature dependencies of ε′ extrapolated from Col$_h$ and Iso phases.

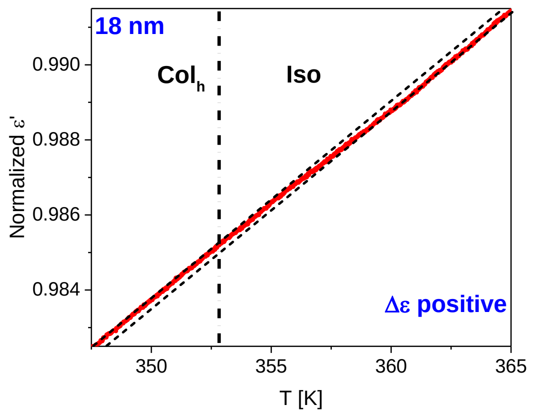

Figure S7. Normalized dielectric permitivities as a function of temperature during the third heating run for the sample with a pore size of 18 nm with unmodified pore walls. The dashed-dotted lines indicate the Col_h-Iso phase transition temperatures determined by DSC. The dashed lines represent temperature dependencies of ε′ extrapolated from Col_h and Iso phases.

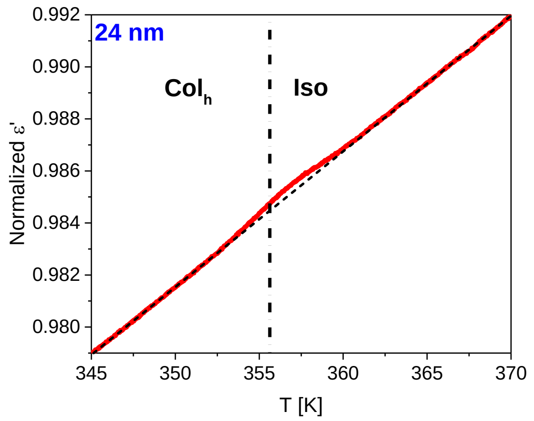

Figure S8. Normalized dielectric permitivities as a function of temperature during the third heating run for the sample with a pore size of 24 nm with unmodified pore walls. The dashed-dotted lines indicate the Col_h-Iso phase transition temperatures determined by DSC. The dashed lines represent temperature dependencies of ε′ extrapolated from Col_h and Iso phases.

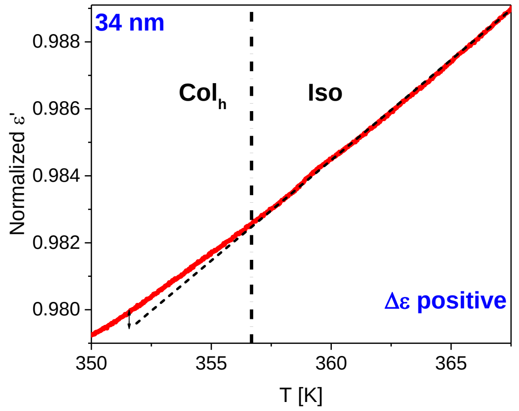

Figure S9. Normalized dielectric permitivities as a function of temperature during the third heating run for the sample with a pore size of 34 nm with unmodified pore walls. The dashed-dotted lines indicate the Col$_h$-Iso phase transition temperatures determined by DSC. The dashed lines represent temperature dependencies of ε′ extrapolated from Col$_h$ and Iso phases.

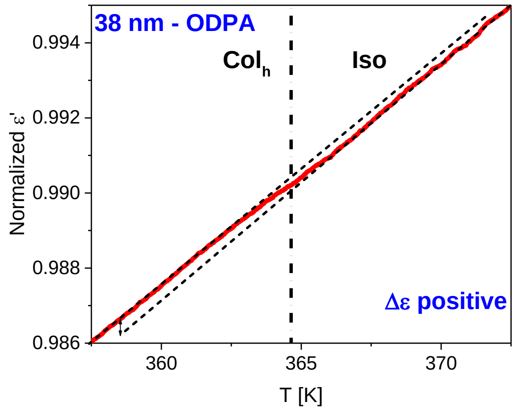

Figure S10. Normalized dielectric permitivities as a function of temperature during the third heating run for the sample with a pore size of 38 nm with modified pore walls. The dashed-dotted lines indicate the Col$_h$-Iso phase transition temperatures determined by DSC. The dashed lines represent temperature dependencies of ε′ extrapolated from Col$_h$ and Iso phases.

REFERENCES

[1] J. Filik, A. W. Ashton, P. C. Y. Chang, P. A. Chater, S. J. Day, M. Drakopoulos, M. W. Gerring, M. L. Hart, O. V. Magdysyuk, S. Michalik, A. Smith, C. C. Tang, N. J. Terrill, M. T. Wharmby and H. Wilhelm, *J. Appl. Crystallogr.*2017, 50, 959–966.

[2] B. R. Pauw, A. J. Smith, T. Snow, N. J. Terrill, A. F. Thünemann, *J. Appl. Crystallogr.*2017, 50, 1800–1811.
